# Juvenile Paget disease with unique compound heterozygous sequence variants in the *TNFRSF11B* gene

**DOI:** 10.1186/s13023-025-03804-2

**Published:** 2025-08-07

**Authors:** Jana Horackova, Renata Taslerova, Milan Bayer, Jana Nekvindova, Ladislava Pavlikova, Jan M. Horacek, Vladimir Palicka

**Affiliations:** 1 Department of Clinical Biochemistry and Diagnostics and Osteocenter, University Hospital Hradec Kralove and Faculty of Medicine in Hradec Kralove, Charles University, Sokolska 581, 500 05 Hradec Kralove, Czech Republic; 2 Department of Pathology, University Hospital Brno, Brno, Czech Republic; 3 Pediatric Osteology, Department of Children and Adolescents, Third Faculty of Medicine, Charles University and Faculty Hospital Kralovske Vinohrady, Prague, Czech Republic; 4 Department of Military Internal Medicine and Military Hygiene, Faculty of Military Health Sciences, University of Defence, Hradec Kralove, Czech Republic

**Keywords:** Juvenile Paget disease, Osteoprotegerin, *TNFRSF11B*, Sequence variant, Mutation, Hyperphosphatasia

## Abstract

**Background:**

Juvenile Paget disease (JPD) is a rare autosomal recessive bone disease characterized by escalated bone metabolism leading to skeletal deformities, susceptibility to fractures, and some extraskeletal findings. This genetic disease is associated with changes in the *TNFRSF11B* gene encoding osteoprotegerin, an important regulator of osteoresorption. Most published JPD cases have been found to carry homozygous *TNFRSF11B* variants, while compound heterozygous variants in this gene have been reported only twice.

**Methods and results:**

We report the first case of JPD diagnosed in the Czech Republic, who presented with a mild phenotype of this disease. The first bone fractures, appeared at 3 years of age. Other clinical manifestations included typical skeletal deformities, macrocephaly, arched chest, lower extremity valgosity, lateral bowing of the thighs, and anterior bowing of the shins. Minor mixed hearing impairment, angioid stripes of the choroidea, and temporary immunodeficiency were present among extra-skeletal findings. Sanger sequencing was performed on both the patient and the parents to test for the presence of *TNFRSF11B* sequence variants. Molecular genetic analysis showed unique compound heterozygous sequence variants in *TNFRSF11B*: a paternally inherited variant c.30 + 5G > A, p.(?) and a maternally inherited variant c.329G > T, p.(Gly110Val). Both of the variants were analyzed by several in silico predictive tools indicating, for their strongly supported pathogenicity according to *American College of Medical Genetics and Genomics* standards. Furthermore, we present diagnostic findings, their treatment, and follow-up care.

**Conclusion:**

The newly described variants of *TNFRSF11B* extend knowledge of this very rare disease. Early diagnosis and antiresorption treatment prevent further fractures and deformity progression, and improve the patient’s quality of life. This example of osteoprotegerin deficiency may help us better understand its role in skeletal and non-skeletal systems.

## Introduction

Juvenile Paget disease (JPD; properly called Paget disease of bone 5, juvenile-onset, MIM #239000) is a rare autosomal recessive genetic disorder characterized by accelerated bone turnover rate, elevated alkaline phosphatase (ALP) level, fragile bones with deformities, and macrocephaly.

Approximately 80 cases of JPD have been described worldwide since 1956 [1–3]. The initial symptoms of JPD occur during infancy and childhood, and affect the entire skeleton, unlike the focal lesions in adult Paget disease of bone (PDB, the second most common bone disease) manifesting itself by hyperphosphatasia.

The early manifestations of JPD consist in skeletal disorders characterized by growth impairment, abnormally widened bones of the extremities, fracture susceptibility, progressive long bone deformities, vertebral compression fractures leading to spinal deformity, skull enlargement, chest deformities (e.g. pectus carinatum), under-mineralization, and hyperostosis [1]. Chong et al. [2] describe distinct phenotypes (severe, intermediate and mild) based on the age onset of the first deformities, mobility, and height limitations.

Extraskeletal findings appear during late childhood. Most patients exhibit hearing impairment or complete hearing loss, which may be caused by ossicle deformities in the internal ear and/or sensorineural hearing loss due to demyelination and degeneration of the acoustic nerve, caused by osteoprotegerin deficiency. Distinctive progressive retinopathy can also be associated with JPD. Angioid streaks, which are characteristic abnormalities of JPD, may be complicated by choroidal neovascularization and disciform scarring, resulting in vision impairment or loss. Dentition abnormalities may occur, leading to premature teeth loss. Further, vascular calcification, premature atherosclerosis, hypertension, and hyperuricemia during late life have also been described. Hormonal issues, such as hypogonadism, hypothyroidism, and growth hormone deficiency could be linked with hypophysis oppression in some cases. In the most severe cases, premature death during early adulthood was usually caused by pulmonary complications due to chest deformities, infections, and respiratory or heart failure [1].

In the majority of cases, JPD results from recessively inherited osteoprotegerin deficiency caused by homozygous or compound heterozygous loss-of-function variants in the *TNFRSF11B* gene [1, 3, 4]. *TNFRSF11B* has five exons and encodes the secreted glycoprotein osteoprotegerin (OPG), a member of the tumor necrosis factor receptor superfamily. OPG is a soluble factor released from pre-osteoblasts and osteoblasts, which in complex with the osteoclast differentiation factor, also known as receptor activator of nuclear factor-kappa B ligand (RANKL) [3], suppresses bone resorption by inhibiting osteoclast activity. Different phenotypes reflect the degree of disruption of the OPG molecule. The most severe phenotype is caused by loss of the entire gene and the lack of the protein; the milder phenotype is caused by minor changes leading to dysfunction of the protein [5].

Furthermore, in rare cases, JPD-like phenotypes can be associated with an activating mutation in the *TNFRSF11A* gene encoding the signal peptide RANK, although this phenotype is consistent with autosomal dominant inheritance [6].

Predominantly homozygous *TNFRSF11B* variants have been described in association with JPD, whereas only two compound heterozygous *TNFRSF11B* variants have previously been reported [4]. We introduce the first case of JPD diagnosed in the Czech Republic: a 23-year-old male with a mild phenotype of the disease, with unique compound heterozygous *TNFRSF11B* variants. To the best of our knowledge, neither of these variants have been described before.

## Methods

### Clinical data

All clinical data about the patient history were retrieved from electronic medical records of the Department of Pediatrics and Osteocenter of University Hospital Hradec Kralove and Department of Children and Adolescents, Faculty Hospital Kralovske Vinohrady, Prague (Czech Republic). Complete biochemical testing was carried out in the biochemical laboratory according to standard protocols. Diagnostic imaging methods (X-ray, scintigraphy and densitometry) were performed at the specialized departments of the University Hospital Hradec Kralove.

### Molecular genetic analysis

Molecular genetic testing was performed at IFCOR Clinical Laboratories (Brno, Czech Republic). The proband’s genomic DNA was extracted from peripheral blood leukocytes using standard laboratory procedures. PCR primers were designed for the amplification of coding and adjacent non-coding regions of the genes *TNFRSF11B* (5 exons, reference sequence NM_002546.4) and *TNFRSF11A* (10 exons, reference sequence NM_003839.4), using the online tools ExonPrimer (Institute of Human Genetics, Germany) and Primer-Blast (NCBI, USA). The regions of interest were amplified from the proband genomic DNA by PCR using the AmpliTaq Gold® DNA polymerase kit (Life Technologies, USA), and enzymatically purified (Exonuclease I and Shrimp Alkaline Phosphatase, New England BioLabs, Canada) according to standard protocols. Subsequently, the sequencing analysis was performed in a 3130 Genetic Analyzer (Life Technologies, USA) using the same PCR primers. The pathogenicity of the sequence variants found was evaluated using bioinformatic prediction tools.

## Results

### Patient clinical and family history

The proband was born to healthy unrelated parents in 1999 as an only child. The family history revealed arterial hypertension and nephrolithiasis on the part of his father and hypothyroidism on that of his mother. Pregnancy, the perinatal period, and early postnatal development were uneventful. The first fracture occurred at age three. It was a left femoral neck infraction after a minor fall. Another fracture of the left humerus, occurred two months later. The patient had a subtrochanteric fracture of the left proximal femur after another fall at age four. The first anatomical abnormalities appeared at age three, *i.e.,* macrocephaly (head circumference + 2.6 SD) and bigger chest (thoracic index + 2.1 SD). Later, the patient manifested gradual and progressive growth disproportion, *i.e.*, short trunk, longer limbs, larger chest, scoliosis of the spine, and valgosity of the lower extremities, with lateral bowing of the thighs and anterior bowing of the shins.

Length asymmetry in the lower extremities required corrective surgery. The height of the patient was within the normal range for his age (50–75th percentile). A biochemical evaluation revealed increased levels of serum alkaline phosphatase (ALP), reaching 75.75 µkat/L at age four, which is 15 times above the upper limit of normal (ULN). Calcemia and parathormone levels were within the normal range. Very low calcium loss in urine and transient proteinuria were observed. After treatment with alendronate, the progressions of growth disproportionality and bone deformities were stopped at age five, and no other fractures appeared. The patient continued treatment with ibandronate due to bone density downtrend, below the Z-score range according to age and gender, and the rise of ALP. Regarding extraskeletal manifestations, minor mixed hearing impairment and angioid bands of the choroidea were diagnosed in the patient, although these disorders did not affect his quality of life in a significant manner. Immunological deficiency with recurrent respiratory infections during childhood required temporary immunoglobulin substitution therapy, but the patient has now been asymptomatic since 2016 and no longer requires treatment in this regard.

### Biochemical testing results

We took the patient at age 19 into the osteological care from our pediatric colleagues. At that time, he had been without antiresorption therapy for three years. Since the diagnosis of JPD, the patient has been in regular rehabilitation care and is in overall good shape. Physical exercise is a regular part of his life and no other fractures have appeared. During initial examination, in addition to the deformities and disproportions described above, persistent signs of high bone turnover rate were observed: ALP 12.54 µkat/L (6 times ULN), serum bone alkaline phosphatase (BAP) 237.9 µg/L (10 times ULN), procollagen type 1 N-terminal propeptide (P1NP) 1 493 µg/L, beta-crosslaps 2.9 µg/L, hypercalcemia 2.76 mmol/L, low vitamin D (25-OH-vit D) 38.4 nmol/L, hyperuricemia 629 µmol/L, low creatinine 45 µmol/L, slightly increased blood sedimentation rate 26/49. Other parameters, such as levels of magnesium and phosphorus in serum and urine, renal and liver function, levels of parathormone, thyroid hormones and prostate-specific antigen, protein electrophoresis, glucose level, lipidogram, and full blood count were within the normal ranges.

### Diagnostic imaging findings

**Fig. 1:**
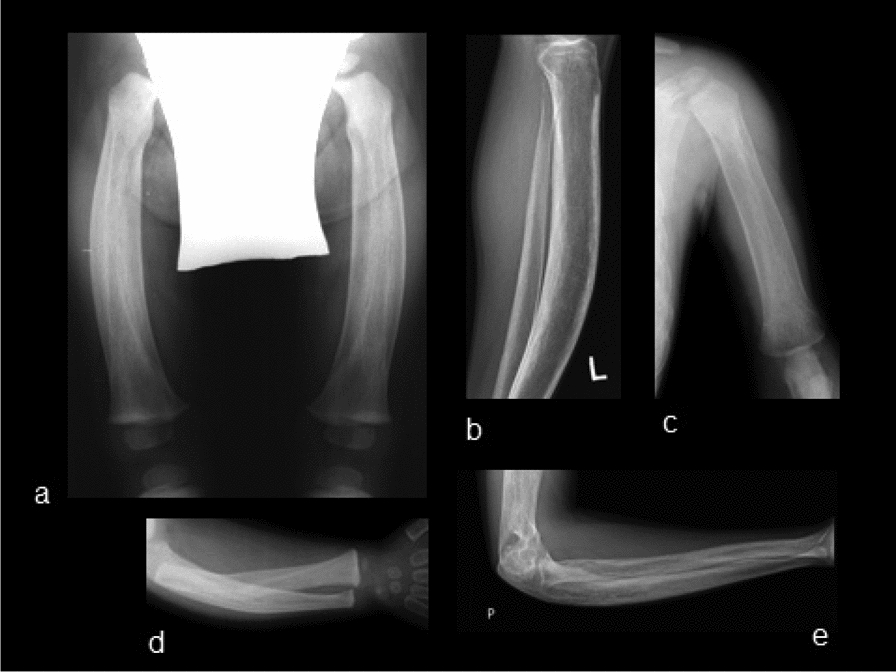
Radiography of long bones (**a** both femurs anteroposterior (AP), age 1 year (Y); **b** left tibia lateral, age 22 Y; **c** left humerus AP, age 1 Y; **d** right forearm AP, age 1 Y; e: right forearm lateral, age 22 Y). All radiographs reveal a diffuse modeling disorder with diaphyseal and metaphyseal undertubulation due to an extremely thickened cortical bone composed of widespread smooth periosteal appositions (featuring a “bone in bone” image), with a various degree of deviation. Diffuse osteosclerosis and a lesser degree of trabecular coarsening can be seen. Epiphyses have been spared

**Fig. 2:**
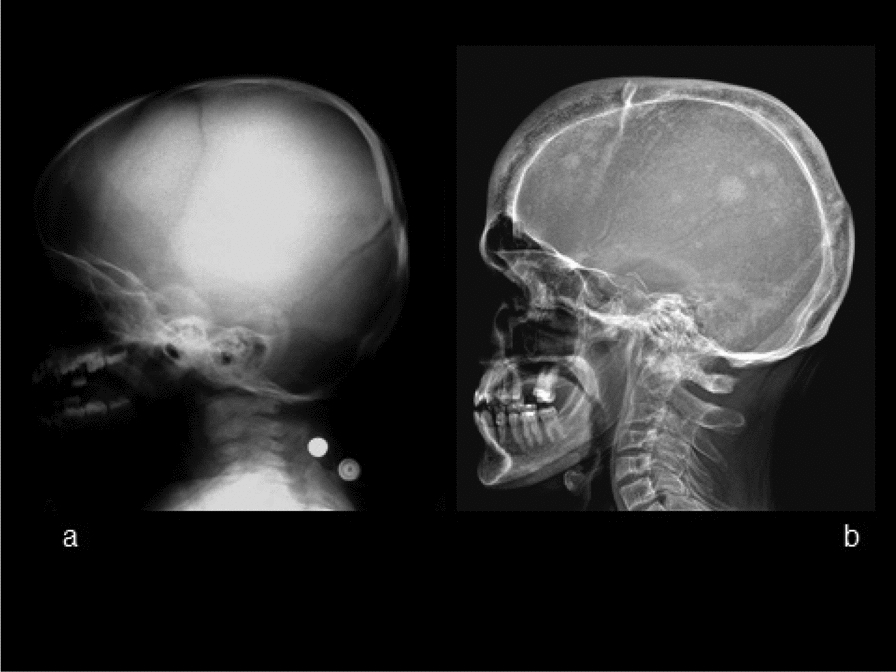
Lateral radiography of the skull and a part of cervical spine (a: age 1Y; b: age 21Y). The radiograph in infancy reveals diffuse osteosclerosis of the skull—both of the calvarium and of the base, without a prominent widening yet. The radiograph in early adulthood reveals marked widening of the skull with periosteal appositions both on the internal and external lamina, bossing of the frontal sinus, diffuse osteosclerosis as well as „cotton-wool “lesions of focal osteosclerosis. Discrete compressions of C3–C5 vertebral bodies are noticeable in the partially shown cervical spine. The vertebral body of C6 has the structure of coarse vertical trabeculae slightly framed by a thickened cortical bone (the image of a „framed vertebra”)

**Fig. 3:**
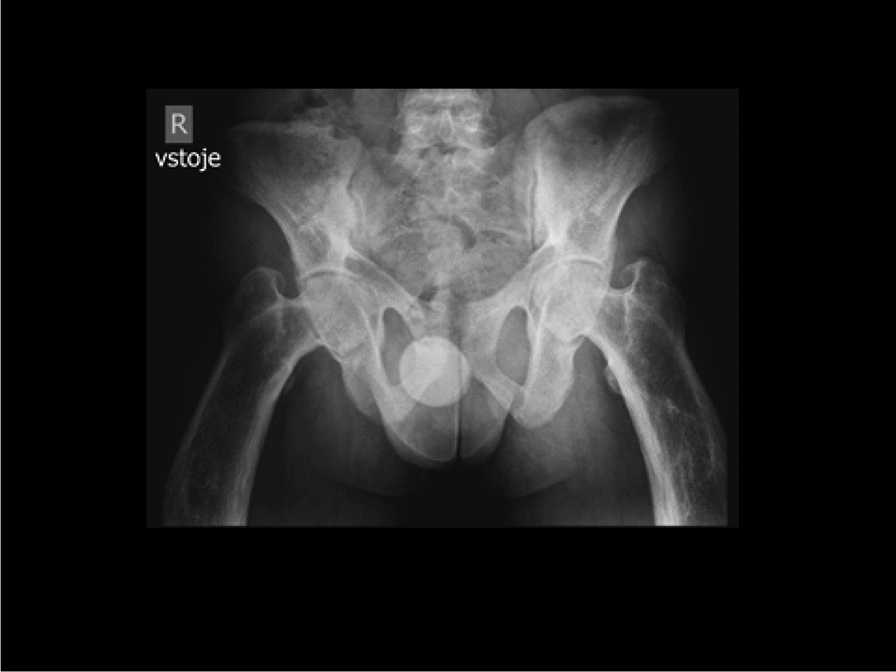
AP radiography of the pelvis (age 20 Y). Diffuse osteosclerosis of the pelvis and both femurs, bilateral acetabular protrusion and varus deformity of both femoral necks can be seen. The proximal diaphyses of femurs are widened due to widespread periosteal appositions, with coarse trabeculae of the spongy bone and with varus deviation

The first skeleton radiographs taken in the autumn of year 2000 were essential for the diagnosis, as they revealed a diffuse modeling disorder in long bones, presenting as extremely thickened cortical bone composed of widespread smooth periosteal appositions (featuring a “bone in bone” image) with varying degrees of deviation. Diffuse osteosclerosis and a lesser degree of trabecular coarsening could be observed (Fig. 1A–E). Diffuse osteosclerosis of the calvarium and the base of the skull during infancy progressed into a marked widening of the skull with periosteal appositions in adulthood. There were also “cotton-wool” lesions of focal osteosclerosis (Fig. 2A, B). Diffuse osteosclerosis of the pelvis and both femurs, bilateral acetabular protrusion, and varus deformity of both femoral necks could be observed in the radiographs. The proximal diaphyses of both femurs were widened with coarse trabeculae of the spongy bone and varus deviation (Fig. 3). Radiographs of the left knee showed diffuse osteosclerosis and a mismatch of the normally shaped epiphyses with extensively widened meta/diaphyses (typical for JPD). Discrete arthrotic changes with emerging osteophytes at the edge of the joint space could be detected in early adulthood radiographs.

Skeletal scintigraphy confirmed increased bone remodeling throughout the skeleton, including the skull, which is consistent with systemic metabolic bone disease. By contrast, it ruled out a focal bone lesion.

Dual-energy X-ray absorptiometry (DXA) images revealed a Z-score of -2.3 in the spine, which is below the normal age and gender range. Further, a trabecular bone score (TBS) of 1.159 showed a significantly impaired bone microarchitecture, which improves fracture risk prediction.

### Molecular genetic testing

**Fig. 4:**
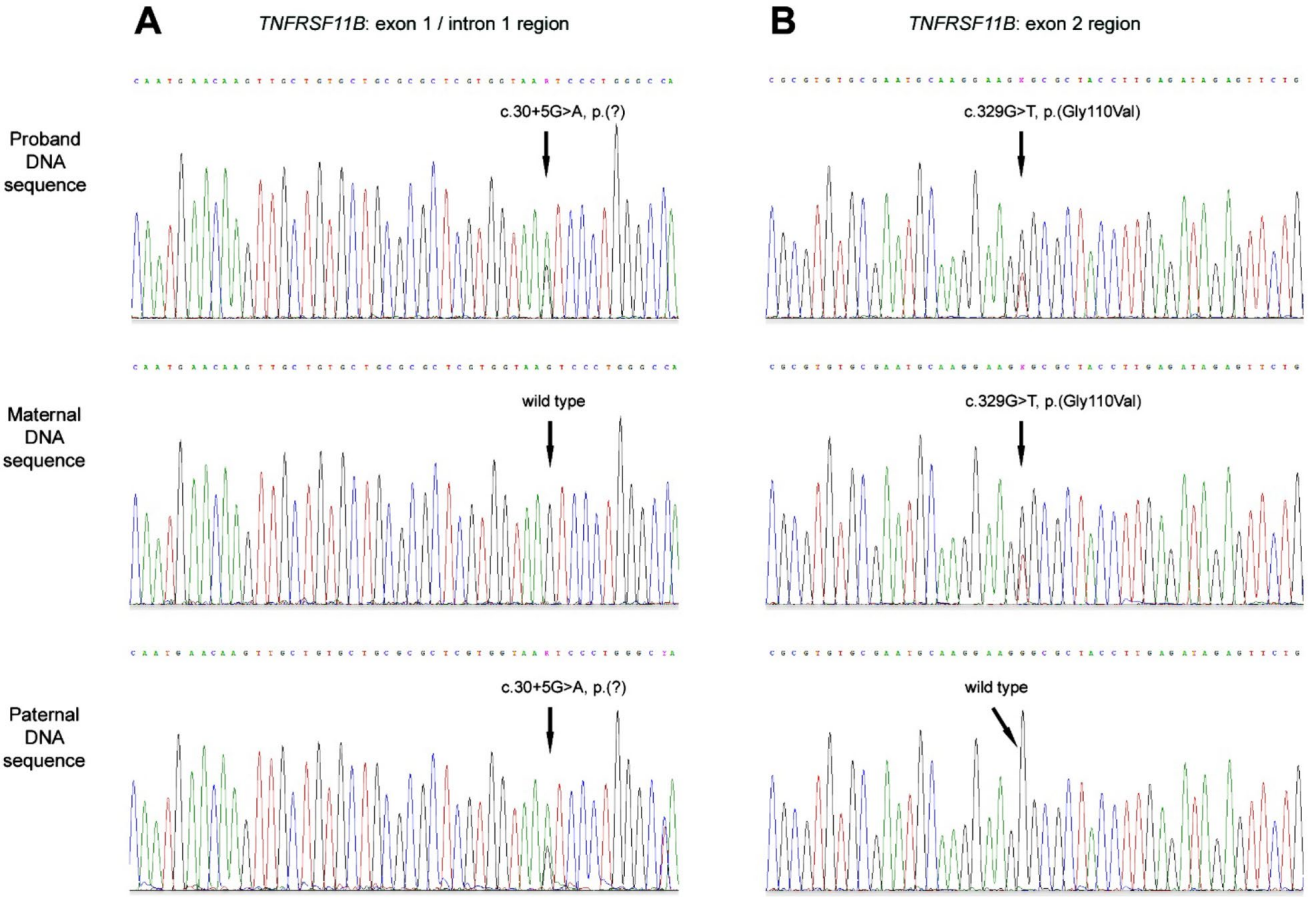
Electropherograms showing mutational and segregation analyses of *TNFRSF11B*. The vertical arrows indicate the location of the mutation events. **A** *TNFRSF11B* exon1/intron1 region: the variant c.30 + 5G > A is present in the proband and paternal DNA sequence (two peaks at one position indicate the presence of two alleles—wild-type G and mutant allele A), while the maternal DNA sequence is wild-type (one peak indicates the presence of one allele—wild-type G). **B** *TNFRSF11B* exon2 region: the variant c.329G > T, p.(Gly110Val) is present in the proband and maternal DNA sequence (two peaks at one position indicate the presence of two alleles—wild-type G and mutant allele T), while the paternal DNA sequence is wild-type (one peak indicates the presence of one allele—wild-type G)

The clinical diagnosis of JPD was highly suspected, and therefore a molecular genetic analysis of *TNFRSF11B* and *TNFRSF11A* genes was indicated in the proband. The sequencing data showed two known polymorphisms and two unknown heterozygous sequence variants in the *TNFRSF11B* gene. No pathological or unknown sequence variant was found in the *TNFRSF11A* gene. A molecular genetic analysis of parental DNA was performed to validate the biallelic localization of the unknown *TNFRSF11B* variants, confirming a paternally inherited sequence variant c.30 + 5G > A, p.(?) and a maternally inherited sequence variant c.329G > T, p.(Gly110Val), neither of which have been previously reported (Fig. 4).

The pathogenicity of both of the variants was evaluated by bioinformatic prediction tools. The sequence variant c.30 + 5G > A leads to a single-nucleotide exchange G for A at position + 5 in the 5´ splice donor site of intron 1. The variant was subjected to VarSome premium 11.18 software (Saphetor SA, Switzerland), which is a powerful tool for clinical characterization of variants. VarSome premium regarded the predictive analyses of three independent bioinformatics tools (Database Splicing Consensus Single Nucleotide Variant, MaxEntScan and Combined Annotation Dependent Depletion), implementing ACMG and AMP standards. According to the ACMG, the variant was classified as PP3, supporting pathogenicity (PP3 definition: multiple lines of computational evidence support a deleterious effect on the gene or gene product). Other bioinformatic tools (Gene Splicer and Splice Site Finder) integrated in the Alamut Visual 2.11 software (Interactive Biosoftware, France) predicted a 30% change in RNA splicing (changes higher than 25% are considered significant), as well as did the Pangolin program, although the 0.05 delta-score of the SpiceAI tool indicated a non-pathological effect of the variant.

The missense variant c.329G > T, p.(Gly110Val) in exon 2 was also determined as pathogenic (PP3 according to the ACMG classification) using the VarSome premium 11.18 software taking into account results from 28 different bioinformatic predictive tools, since Gly110 is a highly conserved amino acid within the functional protein domain.

The pathogenicity of both of the variants is strongly supported by most of the participating prediction programs, as well as by the expressed phenotype of the proband and the positive segregation analysis of the parents. The patient's diagnosis of JPD was thus confirmed by clinical, biochemical, and genetic findings.

### Treatment and follow up

The treatment with alendronate and ibandronate in infancy stopped the progression of bone deformities and prevented further fractures. The antiresorption treatment was again indicated in adulthood after four years of drug holiday, because of a high risk of fractures and deformity progression according to Z-score, TBS score, and bone turnover rate. The therapy was started after dental treatment. Taking into account experience and patient preferences, a peroral form of ibandronate was chosen. There was a significant improvement in laboratory bone turnover parameters—reduction of P1NP by up to 80% (268.2 µg/L), beta-crosslaps by up to 41% (1.71 µg/L), ALP by up to 73% (3.33 µkat/L), and BAP by up to 75% (60 µg/L). Furthermore, the level of vitamin D was normalized after substitution; hyperuricemia was also normalized without medication. However, a mild tendency towards hypercalcemia still persists.

Currently, the patient is pain-free, physically active, and no other fractures have appeared. He is a university student, and has played piano for years, which indicates above-average intelligence and non-significant limitations in hearing capacity. Since the diagnosis requires a multidisciplinary approach, the patient remains under the care of an osteologist, orthopedist, otorhinolaryngologist, endocrinologist, ophthalmologist, and immunologist.

## Discussion

JPD is an example of OPG deficiency in humans [5], as it leads to skeletal and extra-skeletal consequences. In addition to deafness, retinopathy, and premature dental loss, there are other important systemic manifestations.

Osteoprotegerin (OPG) was identified in 1997 as a glycoprotein regulating bone resorption [7]. OPG belongs to the tumor necrosis factor receptor superfamily, and is a decoy receptor for RANKL: through the RANKL/OPG/RANK/NF-kappa B signaling pathway, it inhibits osteoclastogenesis [1, 7]. However, OPG influences also other biological functions such as cardiovascular, immune, and endocrine [7, 8]. Other OPG ligands, e.g. TRAIL (Tumor necrosis factor Related Apoptosis-Inducing Ligand), glycosaminoglycans, proteoglycans, and von Willebrand factor/factor VIII complex, are involved in vascular, immune, and tumor pathophysiology [9, 10].

OPG-deficient mice display an early onset of osteoporosis and arterial calcification [1, 8], suggesting that OPG inhibits vascular calcification. This observation was supported in later studies on animal models, where OPG seemingly acts as a protective factor against calcification [9]. These observations are consistent with the increased incidence of pseudoxanthoma elasticum and vascular calcifications described in JPD-diagnosed patients with OPG deficiency [1]. In contrast, the levels of OPG in serum increase with vascular coronary artery disease, stroke, and cardiovascular events [7–9]. In this regard, the levels of OPG in serum are considered as an independent risk factor in the progression and incidence of cardiovascular diseases, being significantly correlated with their severity and 10-year progression of carotid artery disease, vascular morbidity, and mortality [8]. Therefore, serum OPG level could be used as a promising biomarker of cardiovascular disease. Some studies suggest that serum OPG levels increase in response to vascular insults and inflammation as a compensatory mechanism while others claim that OPG might be a pro-atherogenic factor promoting inflammation and fibrosis [7, 9]. In contrast, TRAIL ligand is a protective factor against atherosclerosis, possibly by inducing the apoptosis of macrophages and vascular smooth muscle cells. It is not clear whether OPG is a risk factor or only a risk marker of cardiovascular disease [7, 8]; therefore, further research is needed in this regard, as it could determine whether recombinant OPG can be used in the treatment of JPD. Our patient does not show any clinical or ultrasound signs of premature atherosclerosis. However, because of his young age and the mild phenotype of the disease it is important to monitor the presence of other risk factors and clinical manifestations of atherosclerosis in the future.

OPG plays a regulatory role in the inflammation process and the immune system. The secretion of RANKL by activated T-cells induces osteoclastogenesis, which may be enhanced by cytokines such as TNF-alpha, IL-1, and IL-17, promoting both inflammation and bone resorption. RANKL activation has been associated with the pathogenesis of rheumatoid arthritis and osteoporosis. Conversely, this system is blocked by OPG, IL-4, and IL-10, which inhibit both inflammation and osteoclastogenesis. OPG is also involved in B-cell maturation and antibody secretion [8]. Our patient presented with childhood immunodeficiency, with recurrent infections of the respiratory tract requiring temporary immunoglobulin substitution. This could be related to OPG deficiency. The need for immunoglobulin substitution was terminated after five years of ibandronate therapy at the age of 16. Relevant association between bisphosphonate therapy and immunodeficiency has not yet been described in JPD patients. A causal relationship seems unlikely due to the high affinity of bisphosphonates for bone mineral (they bind almost exclusively in bone or calcified tissues) [11]. However, there are studies describing a beneficial effect of ibandronate also on serum OPG levels in postmenopausal women [12]. This association could be an explanation for the immunodeficiency improvement after ibandronate treatment, which is certainly only possible in patients where OPG function is at least partially preserved. This would probably not be possible in severe homozygous forms of osteoprotegerin deficiency. Further research in this area would be necessary to clarify the association.

The association between homozygous *TNFRSF11B* variants and JPD was first described by Whyte et al. in 2002 [3]. To date, twenty JPD-causing variants have been described in the *TNFRSF11B* gene (HGMD® Professional release 2024.2). The majority of JPD cases present homozygous sequence variants in this gene; however, there are reports of two JPD patients having compound heterozygous variants in this gene [4, 13]. Our patient presents a relatively mild JPD phenotype caused by two novel sequence variants in a unique and heretofore unreported *TNFRSF11B* genotype: paternal variant c.30 + 5G > A, p.(?) and maternal variant c.329G > T, p.(Gly110Val).

The novel point mutation c.30 + 5G > A in the splice donor site of intron 1, resulting from a G to A substitution in position + 5, was strongly suggested to cause aberrant RNA splicing by most of the prediction tools used. The moderately conserved G to A substitution at the + 5 position of the 5' splice site of different introns has been previously described in various genes. Transcriptome analyses have revealed the pathogenic capacity of + 5 G to A variants. These variants often abolish the consensus splice donor site, leading typically to skipping the entire preceding exon [14–18] or activating a cryptic splice donor site either in the preceding exon or further downstream in the intron [19, 20]. Various combinations of both have also been observed [21, 22].

The variant c.329G > T in exon 2 of the *TNFRSF11B* gene results in a glycine to valine substitution at position 110 of the OPG amino acid sequence. This missense variant was bioinformatically predicted to be most likely pathogenic. Chong et al. [2] have described several missense variants in exon 2 of the *TNFRSF11B* gene, predicting the effects of the identified variants based on knowledge of the structure and functional domains of the OPG homodimer. Each OPG monomer has four N-terminal cysteine-rich domains (CRD I–IV) that behave as independent structural modules, forming two or three disulfide bonds through four or six cysteine residues. The CRD II and CRD III domains are crucial for RANKL binding [23]. This binding interface is composed of two binding sites. The binding site I consists of relatively small and separate contact patches in the OPG “50 s loop” of the CRD II domain (His47, Tyr48, Tyr49, Ser56, Asp57, Glu58, Tyr61, Pro64, Val65, Leu69), while the binding site II consists of continuous patches in the OPG “90 s loop” (Arg90, Leu92, Glu93, Ile94, Glu95, Phe96, Cys97, Leu98, Glu116) of the CRD III domain [23]. Exon 2 encodes amino acids 11–123, and thus includes the entire CRD I, CRD II, and a large proportion of CRD III. The Gly110Val substitution is located to CRD III, very close to the 90 s loop binding site II of OPG as well as close to the disulfide bond formation. According to the findings of Chong et al. [2], there are two possible consequences of this substitution. Either it could affect the ability of the adjacent Cys118 residue to form the disulfide bond or it could change a conformation of the CRD III domain. Both disulfide bond abolition and conformational changes affect the stability of the osteoprotegerin CRD III, which is critical for RANKL binding.

The treatment of JPD is aimed at excessive osteoresorption, for which different kinds of bisphosphonates have been used, e.g. etidronate, pamidronate, alendronate, risedronate, ibandronate, and zoledronic acid [1]. The latter is the most efficient in reducing serum ALP and has a more sustained effect, similarly as for adult Paget disease of bone. Bisphosphonates remain the most widely used therapy for JPD today, which in clinical practice have a favorable effect on the development of the disease. However, some experimental studies on OPG-deficient mice showed a reduced zoledronate effect on bone compared with that on wild mice or with another disorder of the RANKL signaling pathway [24]. The different response to such bisphosphonate therapy is probably influenced by the degree of OPG damage. In the case of better preserved function of OPG, the effect of bisphosphonates can be more effective than in cases with a complete absence of functional OPG. Various phenotypes of homozygous TRNFS11B mutations have been described [2]. Bisphosphonate therapy improves clinical, radiographic, and histological findings. The remission period is influenced by both the potency and duration of the bisphosphonate action, being longer with ibandronate or zoledronic acid. Although bisphosphonates are expected to influence the skeletal manifestations of JPD, retinal lesions and hearing loss may progress despite long-term bisphosphonate therapy [1]. Although there are cases of mild hearing improvement with ibandronate therapy, experimental treatment with risedronate and zoledronic acid in OPG knockout mice resulted in a protective effect on the conductive and sensorineural components of hearing [1, 25, 26].

Treatment with RANKL monoclonal antibodies (denosumab) is currently expanding, as it shows good results on the reduction of ALP, bone pain, and deformities; however, no clear influence on retinopathy or hearing loss in adults has been observed and its safe use on children has yet to be verified [1]. The hope for the future is recombinant OPG therapy, which is not yet available for clinical practice.

## Conclusion

The correct diagnosis and early therapy of JPD significantly affects the prognosis of young patients. Timely commencement of therapy can prevent fractures, bone deformities, and reduced mobility. The clinical report of the first patient diagnosed in the Czech Republic with novel compound heterozygous variants in the *TNFRSF11B* gene may help to improve our knowledge of this very rare disease. JPD cases with OPG deficiency could also improve our knowledge of the role of OPG in skeletal, cardiovascular, metabolic, and immunological pathophysiology.

## Data Availability

All original clinical data are available upon reasonable request from the corresponding author due to their sensitivity. The molecular genetic experimental data and the simulation results that support the findings of this study are available in Zenodo with the identifier 10.5281/zenodo.13341336.
